# To Trap a Pathogen: Neutrophil Extracellular Traps and Their Role in Mucosal Epithelial and Skin Diseases

**DOI:** 10.3390/cells10061469

**Published:** 2021-06-11

**Authors:** Carolina Domínguez-Díaz, Gael Urait Varela-Trinidad, Germán Muñoz-Sánchez, Karla Solórzano-Castanedo, Karina Elizabeth Avila-Arrezola, Liliana Iñiguez-Gutiérrez, Vidal Delgado-Rizo, Mary Fafutis-Morris

**Affiliations:** 1 Doctorado en Ciencias Biomédicas, Con Orientaciones en Inmunología y Neurociencias, Universidad de Guadalajara, Sierra Mojada 950, Guadalajara 44340, Mexico; carolina.dominguezd@alumnos.udg.mx (C.D.-D.); gael.varela@alumnos.udg.mx (G.U.V.-T.); german.munoz2599@alumnos.udg.mx (G.M.-S.); 2 Centro Universitario de Ciencias de la Salud, Universidad de Guadalajara, Sierra Mojada 950, Guadalajara 44340, Mexico; karla.solorzano1972@alumnos.udg.mx (K.S.-C.); karyelinuta@gmail.com (K.E.A.-A.); Liliana.Iniguez@alumno.udg.mx (L.I.-G.); vidalrizo@gmail.com (V.D.-R.); 3 Centro de Investigación en Inmunología y Dermatología (CIINDE), Calz del Federalismo Nte 3102, Zapopan 45190, Mexico

**Keywords:** neutrophils, neutrophil extracellular traps, infection, inflammation, mucosal epithelium, skin disease, inflammatory bowel disease, COVID-19, gonorrhea, systemic lupus erythematosus

## Abstract

Neutrophils are the most abundant circulating innate immune cells and comprise the first immune defense line, as they are the most rapidly recruited cells at sites of infection or inflammation. Their main microbicidal mechanisms are degranulation, phagocytosis, cytokine secretion and the formation of extracellular traps. Neutrophil extracellular traps (NETs) are a microbicidal mechanism that involves neutrophil death. Since their discovery, in vitro and in vivo neutrophils have been challenged with a range of stimuli capable of inducing or inhibiting NET formation, with the objective to understand its function and regulation in health and disease. These networks composed of DNA and granular components are capable of immobilizing and killing pathogens. They comprise enzymes such as myeloperoxidase, elastase, cathepsin G, acid hydrolases and cationic peptides, all with antimicrobial and antifungal activity. Therefore, the excessive formation of NETs can also lead to tissue damage and promote local and systemic inflammation. Based on this concept, in this review, we focus on the role of NETs in different infectious and inflammatory diseases of the mucosal epithelia and skin.

## 1. Introduction

Neutrophils represent 60% of the total leukocyte cells in peripheral blood and form the first defense line in the human body. Furthermore, neutrophils are the first cells recruited to infection sites or inflammation in the tissue. After their maturation in bone marrow, they usually circulate in peripheral blood, between 6 and 10 h. If they transmigrate to vessels and migrate into tissues, they can survive for up to 2 days. Finally, dead neutrophils are eliminated by macrophages. The life of neutrophils depends on the microenvironment and different host factors, such as health, eating habits, the presence or absence of infection and aggressive pathogens [1,2].

These innate immune cells possess at least three different types of granule content: primary or azurophilic granules contain elastase, myeloperoxidase, cathepsin G, defensins and azurocidin for pathogen killing and degradation; secondary or specific granules contain high levels of lactoferrin, pre-cathelicidin, as well as lysozyme and proteins needed for membrane fusion during granule release; tertiary or gelatinase granules contain lactoferrin and metalloproteinases, such as matrix metalloproteinase 9 (gelatinase B), which inhibit bacterial growth through iron sequestration [3]. A fourth type of granule has been described, with a high content in ficolin-1 and a low content in gelatinase, which are rapidly released [4,5]. Secretory vesicles can be also considered among neutrophil granules, and they contain plasma proteins, such as albumin, and cytokines synthesized during immune activation [3].

In the case of injury, neutrophils are responsible for the removal of pathogens and foreign agents; this is possible through three different mechanisms. The first is the release of the neutrophil granule content by exocytosis, which is called degranulation. This process requires two signals: an adhesion-dependent signal involving selectins and the β2-integrin family, and a signal produced by the interaction of immune receptors with their ligands, such as G-protein-coupled receptors (GPCRs), Fcγ receptors and pattern recognition receptors (TLRs). This triggers signaling pathways that lead to the recruitment of granular vesicles to the target membrane, followed by the release of their vesicular content, e.g., cytokines, metalloproteinases, lactoferrin and hydrolytic enzymes, to the extracellular space [3]. However, this can damage other neighboring cells [6,7,8].

Another mechanism that neutrophils employ against pathogens is phagocytosis; a neutrophil is recruited by chemotaxis, followed by the recognition of antigens on the pathogen surface, and finally the uptake of the foreign pathogen, which is mediated by oxygen-dependent or -independent pathways [2]. A failed phagocytosis can occur if the particle or pathogen is too large to be engulfed by the neutrophil, which in turn provokes the release of the granular content of the neutrophil into the extracellular space [9].

Neutrophil extracellular traps (NETs) [10] that act as DNA-peptide-associated networks are the third mechanism by which the neutrophil can remove foreign agents from the body. NETs were first described by Takei in 1996 as a new type of programmed cell death [11] and by Brinkman in 2004 as a microbicidal mechanism involving cell death [10]. The proteins and peptides found within NETs are derived from neutrophil granules, such as elastase, myeloperoxidase and α-defensin; these peptides possess antimicrobial activity, and once this content is released into the extracellular space, the NETs immobilize pathogens and eliminate them, which has been proven in both in vitro and in vivo experiments [12,13].

**Figure 1:**
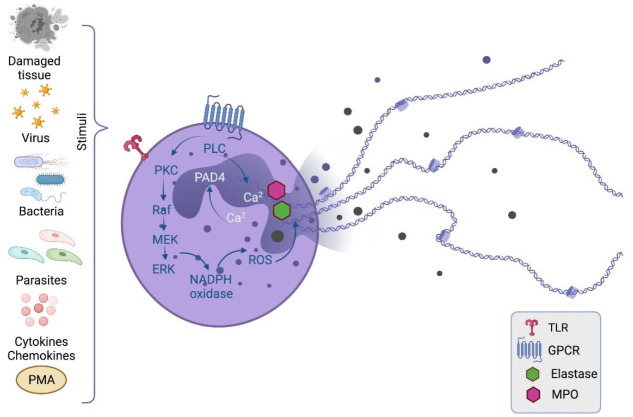
Mechanism of NET generation. NET formation begins with neutrophil activation through the recognition of chemical or biological stimuli, followed by the activation of intracellular enzymes, such as PKC. PKC phosphorylates the Raf-MEK-ERK pathway, where ERK phosphorylates the NADPH oxidase complex, leading to ROS generation. ROS promote the translocation of elastase and MPO to the nucleus where together with PAD4 they lead to chromatin decondensation. Finally, the DNA associated with antimicrobial peptides from the cytosolic granules is released to the extracellular space. PMA, phorbol 12-myristate 13-acetate; PKC, protein kinase C; PLC, phospholipase C; PAD4, peptidyl arginine deiminase-4; ROS, reactive oxygen species; TLR, Toll-like receptor; GPCR, G-protein-coupled receptors; MPO, myeloperoxidase. Created with BioRender.com.

Neutrophils release NETs in response to inflammation or infection (Figure 1). NET formation begins with the activation of the cell in response to the recognition of chemical (Ca^2+^, cytokines, chemokines and PMA) or biological (bacteria, virus, fungi, parasites or damaged tissue) stimuli [12]. Over time, an attempt has been made to classify NET formation according to intracellular mechanisms, phenotype, function and cell vitality. However, the mechanisms are variable according to the stimulus and its quantity, as well as exposure time, so it has been difficult to reach a consensus [14].

The most described NET mechanism involves the activation of signaling pathways via TLRs and G-protein-associated receptors (GPCRs), leading to the activation of protein kinase C (PKC), and PKC phosphorylates Raf kinase, which activates the Raf–MEK–ERK pathway [15]. ERK phosphorylates the NADPH oxidase complex, leading to reactive oxygen species (ROS) production. ROS act as secondary messengers and promote the translocation of elastase and myeloperoxidase from the cytosolic granules to the nucleus [16]. During these events, when the neutrophil is activated, Ca^2+^ is released into the cytosol in response to receptor agonists that activate phospholipase C (PLC). PLC hydrolyzes membrane phosphatidylinositol 4,5-bisphosphate (PIP2), producing the secondary messengers inositol-triphosphate (IP3) and diacylglycerol (DAG), leading to the mobilization of intracellular Ca^2+^ and PKC activation, respectively [17,18].

Regarding the DNA release from the nucleus for the formation of the extracellular trap, it has been proposed that it is a mechanism dependent on the nuclear enzyme protein-arginine-deiminase-4 (PAD4) whose cofactor Ca^2+^ is responsible for the conversion of arginine amino acids (positively charged) into citrulline amino acids (neutrally charged) in histones. This alteration in the amino acid charge weakens the DNA–histone interactions [19]. On the other hand, the elastase and myeloperoxidase activity in the nucleus degrades histones [16]. Both mechanisms lead to chromatin decondensation, leading to the release of the contained energy and consequently the rupture of the nuclear membrane [14].

Finally, the neutrophil releases the DNA into the extracellular space. Various proteins with microbicidal activity are attached to this DNA trap, such as the histones themselves and components of the cytoplasmic granules, as well as elastase, myeloperoxidase, cathepsin G, proteinase 3, cathelicidin (LL-37) and others.

However, although neutrophils have a protective function against infections in the host, as occurs in localized infection sites or in sepsis, their microbicidal response can be excessive and detrimental for the host. For example, in patients with chronic obstructive pulmonary disease (COPD), NETs are associated with increased exacerbation and inflammation [20,21,22]. In addition, in patients with diabetes mellitus, NETs cause tissue damage, as well as impaired wound healing and a prothrombotic state [23,24,25].

Furthermore, NETs have been associated with autoimmune disease. NET formation exposes citrullinated protein autoantigens to the immune system, which contributes to autoantibody production against these DNA–peptide complexes by autoreactive B cells. In turn, these autoantibodies form immune complexes that stimulate NET release in an amplification loop that maintains the inflammatory state in autoimmunity [26]. Evidence of autoantibodies against NET components has been reported in systemic lupus erythematosus (SLE), where autoantibodies against DNA and ribonucleoproteins are found [20,21,27].

There are several reports of chemical stimuli that provoke NET formation. Among the most powerful stimuli, in terms of its intensity in activating the NET formation mechanism, is phorbol 12-myristate 13-acetate (PMA), a protein kinase C activator. Other agents that serve as stimuli are physiological proinflammatory agonists (C5a and IL-8), isolated bacteria (*Streptococcus agalactiae* or Group B *Streptococcus* (GBS), *Staphylococcus aureus*, *Streptococcus pneumoniae*, *Streptococcus pyogenes*, *Escherichia coli*, *Clostridium difficile*, *Shigella flexinheimi*, *Salmonella tyersheimurinheimia*, *Mycobacterium tuberculosis* and *Vibrio cholerae*), viruses (Influenza, Dengue, SARS-COV-2 virus, Human immunodeficiency virus 1 and Respiratory syncytial virus), fungi (*Candida albicans*, *Aspergillus fumigatus* and *Cryptococcus* spp.) and parasites (*Plasmodium falciparum*, *Leishmania amazonensis*, *Eimeria bovis* and *Toxoplasma gondii*). Interestingly, some bacterial strains are able to inhibit NET release, such as *Lactobacillus rhamnosus* [12,27,28].

Since many of these stimuli are microbial in nature, these pathogens enter the body through mucosal surfaces, where neutrophils are the first innate immune cells to confront them. The aim of this review is to explore the role of NETs in different inflammatory and infectious diseases of the mucosal epithelium as well as in the skin.

## 2. NETs in Inflammatory Bowel Disease

Neutrophils play a significant role in the intestinal epithelium maintenance because they prevent bacterial translocation, as well as indirectly promote mucosal wound repair; apoptotic neutrophils in the epithelium promote the macrophage M2 polarization to a repairing phenotype to resolve the inflammation. However, neutrophils also contribute to the inflammatory process in the intestine [29].

Inflammatory bowel disease (IBD) is a chronic condition that results from dysbiosis, which causes an alteration in the immune system [30,31]. IBD is a term that refers to two disorders: ulcerative colitis (UC) and Crohn’s disease (CD). IBD is a complex multifactorial pathology, where genetic and environmental factors predispose individuals to these diseases [32].

UC and CD share some risk factors, such as age, smoking habits, appendicitis, autoimmune diseases, diet, lifestyle and genetics [33]. The *NOD2* gene has been associated with IBD, whose genetic variants are associated with increased susceptibility to the development of CD [34]. Most of the genes shared between UC and CD exacerbate or protect equally, which suggests a similar role in both diseases [35].

UC is an inflammatory bowel disease that occurs gradually or suddenly in the digestive tract; it damages the lining of the colon and rectum, and it affects the mucosa; it is characterized by the presence of a granular mucosa, superficial ulcers, pseudopolyps, diarrhea, abdominal cramps, fever and fatigue, which can cause friability of the mucosa and bleeding from the intestine [33,36]. With regard to CD, the signs are similar; however, the pathology leads to the development of aphthoid ulcers, fistulas, fissures and stenosis [37].

**Figure 2:**
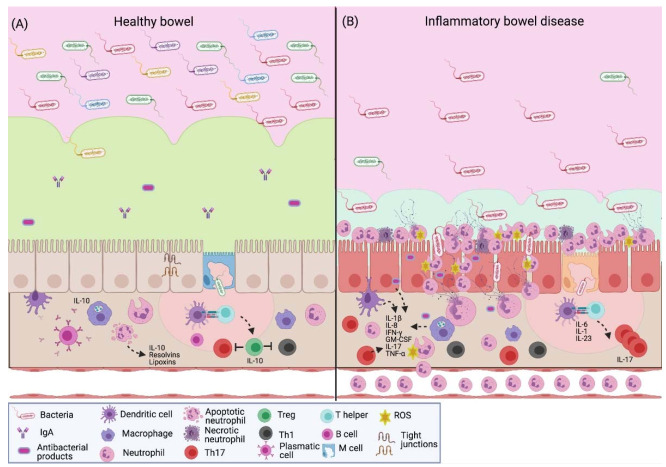
NETs in inflammatory bowel disease. (**A**) The intestinal barrier protects the host against pathogens; it promotes homeostasis between the commensal microbiota and the immune system. In healthy intestinal mucosa, the epithelium is covered with mucus that contains antimicrobial peptides and IgA. In the lamina propria, immune cells are strategically distributed to initiate a response. There is a constant antigen presentation to establish immune tolerance towards the gut microbiota. Mucosal neutrophils prevent the antigen translocation; their apoptosis promotes mucosal injury repair through the secretion of resolvins and lipoxins; it also leads to the M2-type macrophages, which secrete IL-10 to create a microenvironment where Treg lymphocytes predominate. (**B**) Patients with IBD present dysbiosis and a low expression of tight junction proteins, which leads to an increase in intestinal permeability. Epithelial and immune cells produce proinflammatory cytokines (IL-1β, IL-8, IFN-γ, GM-CSF, TNF-α and IL-17) that increase the transendothelial migration and neutrophil activation to create a microenvironment where the activity of Th17 lymphocytes predominates. Neutrophils respond to luminal antigens through a respiratory burst, degranulation and the release of extracellular traps with antimicrobial factors, such as elastase and myeloperoxidase, which contribute to the characteristic colonic tissue damage in these patients. IgA, immunoglobulin A; IL-1β, interleukin-1β; IL-6, interleukin-6; IL-8, interleukin-8; IL-10, interleukin-10; IL-17, interleukin-17; IL-23, interleukin-23; IFN-γ, interferon gamma; GM-CSF, granulocyte-macrophage colony-stimulating factor; TNF-α, tumor necrosis factor- α; ROS, reactive oxygen species. Created with BioRender.com.

The intestinal epithelium is composed of a single layer of epithelial cells that form a continuous barrier through tight junctions, composed of extracellular proteins as the family of claudins, occludin and tricellulin. These cells together with immune cells maintain a balance between the intestinal microbiota and the host (Figure 2A). Altered claudin and occludin expression has been documented in IBD models. In the epithelium of IBD, the intestinal barrier is compromised; the absence or change in these molecules causes “an exacerbation in the disease severity through the increase in various proinflammatory cytokines, including IL-1β, TNF-α and IFN-γ, secreted by immune cells” [35,38]. In addition, mucosal lesions, increased epithelial permeability, invasion of commensal bacteria in the subepithelial space and massive neutrophil recruitment are observed [39]. The neutrophil response promotes the inflammatory state through the release of NETs, which contribute to the damage of the colonic epithelium (Figure 2B).

An ex vivo study analyzed the presence of NET-associated proteins in colon biopsies from patients with CD, UC or colon cancer. PAD4, myeloperoxidase, neutrophil elastase and citrullinated histone H3 were highly expressed in the pathology of UC compared to CD; the neutrophils related to UC produced more NETs in response to stimulation to TNF-α and an improvement was observed in patients who received anti-TNF-α treatment, where the expression of NET-associated proteins was reduced [40]. These results demonstrate that in UC, a positive interaction between TNF-α and NETs exists, where TNF-α stimulates NET-formation, which in turn promotes TNF-α secretion, inducing the clinical manifestations of the disease. Patients with severe UC benefit from anti-TNF therapy because it controls the inflammatory response and NET formation in the intestinal epithelium.

A cohort study evaluated patients with UC and patients without a diagnosis of IBD through colon biopsy. NETs were correlated with PAD4 expression in the intestinal mucosa, as shown by Western blot experiments. PAD4 stimulates the release of NETs, through the citrullination of histones. This leads to the chromatin decondensation and DNA release. The presence of NETs is observed mainly in the inflamed mucosa of UC [41], suggesting that NET release is correlated with characteristic anatomical damage. Therefore, the neutrophil response has an unfavorable effect on the resolution of the inflammatory state.

In a study of IBD patients, peripheral blood and colon neutrophils were analyzed. It was observed that patients with the active disease present greater NET release compared to patients with the inactive pathology; these in turn present a greater response with respect to healthy subjects. Likewise, it has been reported that patients with UC have a greater NET presence than patients with CD. Furthermore, inefficient degradation of NETs was found in the sera of these patients with the active disease [42], which shows that the lack of control in the neutrophil response leads to a greater epithelial damage and inflammation.

Nonetheless, in an experimental murine model of colitis, it was confirmed that the degradation of NETs is a protective factor against the exacerbated expression of proinflammatory cytokines, the development of tumorigenesis associated with IBD and the generation of thrombosis. In regard to thrombosis, NETs predispose to a prothrombotic state through the release of phosphatidylserine (PS). PS activates TLR2 and TLR4 in platelets and endothelial cells, which induces in these cells a profile that favors coagulation and, therefore, thrombosis [42].

Angelidou et al. also reported a higher NET production in patients with active UC compared to CD and healthy patients. Likewise, a greater presence of the proinflammatory cytokine IL-1β and the tissue factor thromboplastin (TF) was found in NETs obtained from both colonic tissue and blood from these patients. The induction of these NETs is related to autophagy, which is induced by the REDD1 protein [43]. These findings suggest that NETs have an important role in maintaining the inflammatory and thrombogenic environment associated with the clinical characteristics of UC.

This demonstrates that in IBD, particularly in UC, there is a greater release of NETs, which is associated with greater damage to colonic tissue, a characteristic manifestation of this disease, and predisposes the patient to extraintestinal pathologies, such as thrombosis. The use of therapies targeting NET components in IBD has been reported, where inhibitors directed to PAD, elastase and NET-associated DNA reduce the clinical manifestations of these pathologies; however, further studies are needed to evaluate this therapeutic strategy [44].

## 3. NETs and Respiratory Diseases

In 2017, the Forum of International Respiratory Societies (FIRS) reported that respiratory diseases are among the most common causes of severe illness and death worldwide. About 65 million people suffer from chronic obstructive pulmonary disease (COPD), which causes three million deaths per year. With 334 million deaths, asthma is the most common disease in children, affecting 14% of the global infant population. Pneumonia has been catalogued as the main cause of death in children under 5 years old, killing 808 thousand (this represents 15% of all deaths in infants), whereas tuberculosis (TB) is the deadliest infectious disease for humans, affecting over 10 million people, and causes 1.4 million deaths per year [45].

Currently, we can add the coronavirus disease (COVID-19) to this list; it is caused by Severe Acute Respiratory Syndrome Coronavirus 2 (SARS-CoV-2) [46], which to date has infected about 144 million people and killed more than three million worldwide [47]. The effect of NETs in respiratory diseases has been widely studied, and the results indicate that there are more detrimental than protective effects for the host. In this section, we discuss the effect of NETs on the most important respiratory diseases according to the FIRS and the World Health Organization (WHO).

### 3.1. Chronic Obstructive Pulmonary Disease

**Figure 3:**
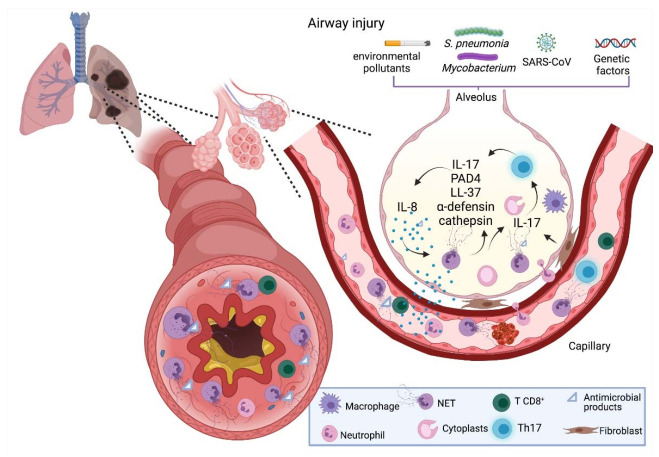
NETs in respiratory diseases. COPD is a multifactor disease caused by physiological and environmental factors (e.g., comorbidities and environmental pollutants) that promote neutrophil recruitment, induce NET formation and contribute to inflammation through LL-37, inducing IL-17 production and hence fibroblast-mediated lung fibrosis. Neutrophilic asthma is mediated by Th17 cells, which cause the upregulation of cathepsin and α-defensin, endogenous production of IL-8 in epithelial cells and induce neutrophil-derived cytoplasts that contribute to Th17 cell differentiation in a PAD4-dependent manner. In pneumonia and TB, NETs have a detrimental role, where they are unable to clear the infection and contribute to the inflammatory state of these pathologies. During Sars-CoV-2 infection, neutrophils are recruited due to a chemokine overexpression (CXCL2 and CXCL8), producing neutrophilia and high NET release, which correlates with disease severity and immunothrombosis. COPD, chronic obstructive pulmonary disease; TB, tuberculosis; NET, neutrophil extracellular trap; IL-8, interleukin-8; IL-17, interleukin-17; PAD4, peptidyl arginine deiminase-4; LL-37, cathelicidin. Created with BioRender.com.

COPD is a multifactor disease caused by early life events; age; physical inactivity; comorbidities; infections; and chronic exposure to stressors, such as tobacco smoke, outdoor pollutant and toxic smoke, which cause neutrophil retention in the airways [48,49]. There is an increased number of neutrophils and NETs in the airways and sputum of COPD patients [50,51], which correlates with the disease severity [20,48,52,53]. It has been demonstrated in ex vivo experiments with peripheral blood neutrophils from patients with COPD that there is elevated NET production compared with healthy controls when stimulated with LPS 10 µM [54]. This inflammatory response contributes to generalized fibrosis mainly through IL-17, which promotes the fibrotic activity of lung fibroblasts [55]. Among the components involved in NET formation, PAD4 is an essential enzyme that plays a key role in chromatin decondensation [19]. Lung tissue samples from COPD subjects present an increased expression of PAD4 at the protein level; the authors proposed that such an increase is due to inflammation [56]. The NET components LL-37 and α-defensin promote the inflammatory response by activating the inflammasome and CXCL8 release from epithelial and smooth muscle cells [57] (Figure 3). Evidence indicates that chronic inflammation generated in COPD promotes neutrophil recruitment, NET release and thus lung injury; however, the role of NETs in COPD patients remains poorly understood, and more studies are needed to elucidate their implications in this disease.

### 3.2. Asthma

Asthma is a chronic disease characterized by inflammation in the respiratory tract and airflow obstruction. It causes intermittent attacks of breathlessness, wheezing and coughing [58]. In recent years, a new phenotype for asthma has been recognized, termed “neutrophilic asthma”, mediated by Th17 cells [59,60]. Patients with neutrophilic asthma present an upregulation of α-defensins, cathepsin G and elastase; this promotes lung inflammation and may exert the release of immature neutrophils into the circulation [61] (Figure 3). Furthermore, patients with neutrophilic asthma are less responsive than eosinophilic asthma patients to corticosteroid treatment [62]. Additionally, ex vivo experiments have shown that NETs are able to stimulate airway epithelial cells and induce endogenous CXCL8/IL-8 production [63], a potent NET inducer [10], which may amplify inflammation [64]. Finally, an in vivo study in a mouse model showed that neutrophils induce complex immune responses to allergens through neutrophil-derived cytoplasts formed during NETosis, which retain functional properties, such as phagocytosis, and induce Th17 cell differentiation in a PAD4-dependent manner, thereby promoting lung neutrophilia [65]. Accordingly, investigations suggest a detrimental role for NETs in asthma; however, more studies are needed to elucidate the main cause of NET formation in this disease.

### 3.3. Pneumonia

Pneumonia is an infection that causes inflammation in alveoli. Community-acquired pneumonia (CAP) is the most common type of infectious pneumonia, and *S. pneumoniae* is the main species of bacteria involved in this disease [66,67]. Ebrahimi et al. found a positive association between high levels of NETs and adverse effects in patients with CAP, such as longer treatment duration, prolonged patient stay, slow recovery and increased mortality, whereas lower levels of NETs were associated with a better outcome, suggesting that NETs may be a reliable biomarker for prognosis in CAP [67]. Added to this, results from in vivo and in vitro studies indicate that NETs do not participate in bacterial killing, but instead contribute to lung damage [68,69]. Another study, performed in a mouse model of severe bacterial pneumonia, showed that NETs were reduced in *PAD4^−/−^* mice when compared with wildtype mice, but had higher bacterial counts in the lungs. The authors also found that *PAD4^−/+^* mice had intermediate NET production and observed an improved survival rate, suggesting that NETs prevent bacterial dissemination in the lung, and a critical balance is necessary to prevent lung injury and maintain microbial control [69].

Despite the protective role of NETs originally proposed by Brinkmann et al. in 2004, it is difficult to confirm such a role for NETs in pneumonia, as they prevent bacterial dissemination but can also cause lung injury. Additionally, bacteria possess evasion strategies to avoid NET-mediated killing, further complicating our understanding of NET release in respiratory infections [70]. These evasion strategies include: (1) endonuclease production (e.g., *S. pneumoniae, S. aureus* and *S. pyogenes*), (2) biofilm formation (e.g., *Pseudomonas aeruginosa* and *Haemophilus influenzae*), (3) antioxidant production (e.g., *H. influenza*), (4) suppression of ROS generation (e.g., *Bordetella pertussis* and *Cryptococcus neoformans*) and (5) virulence factors (e.g., *Burkholderia pseudomallei*) [71,72,73,74], which improve bacterial survival. In this regard, further studies are needed to determine whether NETs have a beneficial or detrimental role in the inflammatory pathologies of the lung.

### 3.4. Tuberculosis

TB is a chronic infectious disease caused by *Mycobacterium tuberculosis*, which mainly affects the lungs and induces a detrimental immune response termed “chronic granulomatous inflammation” [75]. Neutrophils are fundamental in the granulomatous response through CXCR3 signaling [76]. It has been demonstrated that *M. tuberculosis* is able to induce the release of NETs in vitro [77]; however, in an experimental model with Guinea pigs, it was found that NETs are unable to eliminate these bacteria [78]. Furthermore, another study with TB patients revealed that lung tissue damage is associated with neutrophilia and the presence of the NET marker citrullinated histone H3 (cit-H3). Patients with neutrophilia have higher levels of cit-H3 and show no improvement in chest radiograph evaluation after anti-TB treatment, but increased cavity formation. This indicates that NETs play a key role in TB pathogenesis [79]. Finally, it was reported that TB induces the generation of a sub-population of neutrophils termed “low-density granulocytes” (LDGs) that produce high amounts of NETs, and this effect was suppressed by scavenging or blocking ROS [80]. The present information confirms the detrimental effect of NETs in TB by promoting the inflammatory response; however, further research is needed to elucidate the pathologic implications of NETs and evaluate possible therapeutic targets, such as ROS generation, to avoid their excessive formation and, consequently, lung damage.

### 3.5. COVID-19

In December 2019, a rare cluster of pneumonia was reported in citizens from the province of Wuhan, China [81]. This type of pneumonia termed “COVID-19” by the WHO was found to be caused by the SARS-CoV-2 through interaction of the Spike-glycoprotein and the host receptor proteins angiotensin-converting enzyme 2 (ACE2) and the transmembrane protease serine 2 (TMPRSS2), present in the alveolar cells of the lung [82,83]. The symptoms are similar to those of the common cold; however, the main clinical manifestations that distinguish COVID-19 are high fever and dyspnea [84]. Severe cases of COVID-19 present an imbalance between inflammatory and antiviral responses, causing an upregulated expression of chemokines and interleukins [85]. This cytokine storm is characterized by elevated plasma concentrations of IL-2, IL-7, IL-10, GCSF, IP-10, MCP-1, MIP-1A and TNF-α [23,82,86], and contributes to the development of acute respiratory distress syndrome (ARDS) [87].

Several changes occur in immune cell populations during SARS-CoV-2 infection, where neutrophils are recruited throughout the first 4 days [88] due to the overexpression of NF-κB-driven chemokines (e.g., CXCL2 and CXCL8) [89]. Studies have demonstrated that patients with severe disease present high amounts of neutrophils in the blood and lungs [90,91,92]. Additionally, the presence of high levels of NETs in the peripheral blood and lung tissue of patients with COVID-19 has been reported, which correlates with detrimental effects, such as vascular occlusion in the lungs, kidney and liver, and immunothrombosis [85,93,94]. A recent in vitro study that used plasma neutrophils and tracheal aspirate from COVID-19 patients demonstrated that SARS-CoV-2 induces the release of NETs, but this depended on ACE2, serine protease activity, virus replication capacity and PAD4 function [95], indicating that experimental studies with inactivated SARS-CoV-2 may not produce reliable results.

Golonka et al. described that SARS-CoV-2 infection presents two stages: an “early stage” defined by the lack of host antiviral immune responses and a “late stage” characterized by the cytokine storm [96]. Some authors have proposed the use of immunotherapies targeting inflammatory molecules (e.g., IL-1β, IL-6 and TNF-α) in severe COVID-19 cases in order to reduce inflammation and inflammation-associated lung damage, and to prevent admission to the intensive care unit [87]. Considering the stage of the SARS-CoV-2 infection, NET inhibitors (e.g., DNase, disulfiram, inhibitors of neutrophil elastase and PAD4 inhibitors) could aid in the prevention of lung damage [97]. In the first stage, there would be no effect with the use of these inhibitors, but in the “late stage”, it would be able to prevent pulmonary and cardiovascular damage [96].

## 4. NETs in the Genitourinary Tract

The urinary system and genital tract in males and females are referred to together as the genitourinary tract due to their overlap, embryological origin and proximity. The mucosal surfaces of this system are entry points for viral, bacterial, fungal and parasitic pathogens for sexually transmitted diseases (STDs) as well as urinary tract infections (UTIs). Both the epithelial cells lining these structures and the mucosal immune cells have an important role in the defense of this tract and maintain homeostasis with the genitourinary microbiota that colonize these surfaces [98].

### 4.1. Urinary Tract Infections (UTIs)

**Figure 4:**
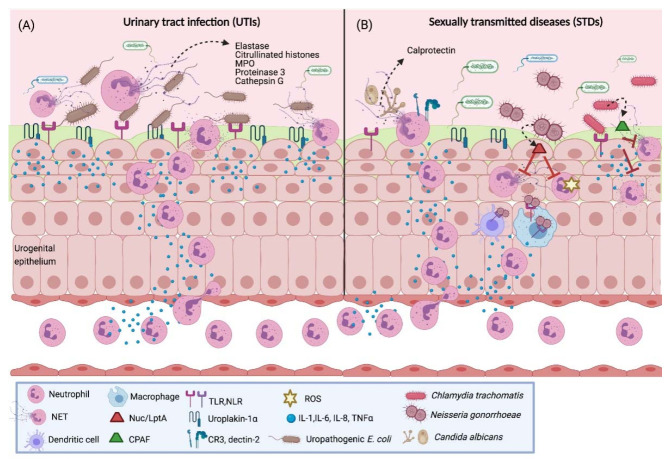
NETs in UTIs and STDs. (**A**) During UPEC infection, bacteria interact with bladder epithelial and immune cells of the urogenital epithelium through TLRs, NLRs and uroplakin-1a, which leads to the production of proinflammatory cytokines that induce neutrophil migration to site. Neutrophils release extracellular traps with antimicrobial peptides, such as citrullinated histones, elastase, MPO, proteinase 3 and cathepsin G, which eliminate the infection. (**B**) In *C. albicans* infection, epithelial cells secrete cytokines that act as neutrophil chemoattractants; neutrophils recognize this pathogen through CR3 or dectin-2 receptors, which induce NET release. Calprotectin, a protein associated with the DNA of extracellular traps, possesses antifungal activity which aids in the infection clearance. However, other bacteria, such as *C. trachomatis* and *N. gonorrhoeae*, have evasion mechanisms characterized by secreting factors, such as the protein CPAF, and the enzymes Nuc and Lpta, respectively, which prevent degranulation and cytokine release, degrade NET components and inhibit ROS-dependent pathways for their release. UTIs, urinary tract infections; STDs, sexually transmitted diseases; NET, neutrophil extracellular trap; MPO, myeloperoxidase; LptA, LOS phosphoethanolamine transferase A; CPAF, Chlamydial Protease-like Activating Factor; TLR, Toll-like receptor; NLR, NOD-like receptor; IL-1, interleukin-1; IL-6, interleukin-6; IL-8, interleukin-8; TNF-α, tumor necrosis factor-α. Created with BioRender.com.

Urinary tract infections (UTIs) are common bacterial infections, particularly among women aged between 15 and 29 years, possibly due to the female anatomy, since the close proximity of the urethra to the vagina and the rectum, and the shorter distance to the bladder, facilitate bacterial colonization. The symptoms of UTI include high frequency and urgency of urination, dysuria and pelvic or abdominal pain. Furthermore, 74.2% of UTI cases are caused by uropathogenic *E. coli* (UPEC), followed by *Klebsiella pneumoniae* (6.2%), *Enterococcus* spp. (5.3%) and *S. agalactiae* (2.8%), among others [99,100]. While these pathogens have evolved mechanisms to evade the host immune response, they can be detected by innate immune cells via fimbriae and flagella by binding to TLRs (TLR4 and TLR5) and proteins, such as uroplakin-1a. This leads to cytokine and chemokine secretion, e.g., IL-8, and neutrophil recruitment (Figure 4A) [100].

A study analyzed urinary pellet samples from UTI patients and found high counts of neutrophils as well as DNA fragments, histones and azurophilic granule effectors similar to in vitro-formed NETs. Evidence of early phase NET formation was observed through both the neutrophil morphology and the proteomic response of uropathogens, such as *S. aureus* and *E. coli*, in the samples, which revealed an adaptive response against neutrophils, with low ribosomal protein synthesis and increased secretion of virulence factors. The proteomic analysis of the urinary pellet samples showed the presence of NET-associated proteins, such as myeloperoxidase, neutrophil elastase, proteinase 3 and cathepsin G, and citrullinated histones [101]. These results indicate that neutrophils participate in the elimination of the pathogens involved in UTIs by NET release; however, further studies are needed to completely understand the interaction between uropathogens and NETs in these infections.

### 4.2. Candidiasis

Candidiasis refers to the most common and opportunistic yeast infections, which can occur in skin, nails and the mucosal tracts of the body; it is caused by *Candida* spp., in particular, *Candida albicans*. Genital candidiasis affects both women and men. While *Candida* yeasts are part of the vaginal microbiota in 20–30% of women, vulvovaginal candidiasis (VVC) affects 75% of women at least once in their lifetime [102,103]. The symptoms include vulvar itching, irritation and pain, dysuria and vaginal discharge. In men, candidal balanitis causes penile redness, irritation, itching and preputial discharge. Among the predisposing factors, diabetes mellitus, the use of broad-spectrum antibiotics, oral contraceptives and vaginal secretions of the sexual partner are associated with a higher incidence of genital candidiasis [104]. Once the balance in the genital microbiota is disrupted, epithelial cells produce cytokines, alarmins and calcium-binding proteins that act as chemoattractants that stimulate neutrophil migration [105,106]. This neutrophil response is responsible for the damage and symptoms produced during the fungal infection [105,107]. However, neutrophils are able to clear the infection through NET formation, whether *C. albicans* is found both in its yeast, as well as in its hyphae form [108]. Depending on whether *C. albicans* is opsonized or unopsonized, it can be recognized by CR3 (CD11b/CD18) or dectin-2 receptors on the neutrophil surface, respectively (Figure 4B). It is also reported that the NET formation mechanism involved is independent of the NADPH oxidase pathway [109,110]. Once it is captured in the NETs, this fungal pathogen is killed by the calprotectin content in the NETs, a key component that gives the NETs their antifungal activity [108,111]. This shows an important role of NET release in the clearance of this fungal pathogen.

### 4.3. Gonorrhea

Nevertheless, some pathogens are able to evade the immune response by neutrophils and survive in the genitourinary tract. Gonorrhea is an STD caused by the obligate pathogen *Neisseria gonorrhoeae*, with an estimated 87 million new cases each year worldwide [112]. Symptoms in men include dysuria, with purulent discharge, and it can result in urethritis; women in general are asymptomatic; however, they can develop cervicitis, and severe complications, such as pelvic inflammatory disease (PID), ectopic pregnancy and sterility, and infant blindness through vertical transmission during childbirth [113]. Both the WHO and CDC have recognized the challenge to prevent and control *N. gonorrhoeae* infection due to the emergence of antimicrobial-resistant strains [114]. The infection induces a strong inflammatory response characterized by neutrophil infiltration recruited by proinflammatory cytokines secreted by epithelial and immune cells that interact with *N. gonorrhoeae* through surface receptors, such as TLRs and NLRs [115].

However, despite the high neutrophil influx to the infection site, these cells are unable to eliminate the pathogen, since *N. gonorrhoeae* evades the neutrophil response through several mechanisms [116]. While gonococci induce NETs through an oxidative burst-independent pathway, the NETs are unable to kill the pathogens [117]. The reason for this could be that *N. gonorrhoeae* has evolved in order to release proteins to defend the pathogen against the different antimicrobial components contained in the NETs (Figure 4B). Nuc is a thermonuclease encoded in the genome of all the *N. gonorrhoeae* strains that degrades the DNA in the NETs; the lack of this protein increases the bacteria killed by the NETs [118]. The gonococci also use the enzyme LptA (LOS phosphoethanolamine transferase A) to modify the lipid A portion of the lipooligosaccharide with the addition of phosphoethanolamine, and this increases their survival against the cationic antimicrobial proteins in NETs [116]. In addition, the MtrCDE efflux pump also contributes to the survival of the pathogen through the export of antimicrobial proteins in NETs [119]. Finally, *N. gonorrhoeae* produces the TonB-dependent transporter TdfH to bind to calprotectin, a component of NETs, in order to use the protein as a zinc source and survive [120]. This demonstrates that the gonococci are able to survive the NET-mediated killing through the production and secretion of proteins that act against the antimicrobial components in the NETs.

### 4.4. Chlamydia Trachomatis

*Chlamydia trachomatis* is an obligate intracellular pathogen that is the most common bacterial cause of STDs, with 127 million each year worldwide [112]. Up to 70–80% of infections with *C. trachomatis* in women and 50% in men are asymptomatic. It can cause urethritis, cervicitis, PID, infertility, ectopic pregnancy and miscarriages in women. Epithelial cells infected with *C. trachomatis* secrete proinflammatory cytokines and chemokines, such as IL-1, IL-6, IL-8, GM-CSF and TNF-α, to recruit innate immune cells to the infected area. Likewise, Th1 cells secrete IFN-γ to activate the innate immune response and clear the infection [121]. However, *C. trachomatis* escapes the host immune response; it prevents neutrophil degranulation, cytokine and chemokine production and NET release (Figure 4B). This pathogen inhibits neutrophil activation through the release of the Chlamydial Protease-like Activating Factor (CPAF), which is a protease that cleaves the neutrophil surface receptor FPR2 (Formyl peptide receptor 2). This inhibits the signaling pathways that lead to the release of NETs and, therefore, the pathogen clearance. A CPAF-mutant *C. trachomatis* strain, which secretes a truncated and inactive isoform of CPAF, is able to stimulate NET production, which corroborates the role of this protease in the immune evasion of the Chlamydia infection [122].

### 4.5. Trichomonas Vaginalis

*Trichomonas vaginalis* is a protist parasite responsible for the highly prevalent infection trichomoniasis, which is the most common non-viral STD with 156 million new cases each year worldwide [112]. While the infection can be asymptomatic, symptoms can appear within 6 months after the initial infection and include urethritis, cervicitis, vaginitis, urethral and vaginal discharge, dysuria and pruritus [123]. Additionally, it is associated with a higher risk of PID development, as well as HIV infection [123,124]. The immune response against this parasite involves the secretion of IL-1, IL-6, IL-8 and IL-17 by the epithelial cells [124]. The recruitment of neutrophils is important for the defense against the infection. Nevertheless, *T. vaginalis* is unable to trigger the release of NETs. In order to eliminate this parasite, neutrophils perform another mechanism, known as trogocytosis, where they take fragments of *T. vaginalis* to kill the pathogen [125].

## 5. NETs and Skin Diseases

NETs have been associated with a group of diseases affecting the skin, which is considered a key component in the pathogenesis of these inflammatory skin diseases. In this section, we examine the role of NETs in SLE, psoriasis and ANCA-associated small-vessel vasculitis.

### 5.1. Systemic Lupus Erythematosus

**Figure 5:**
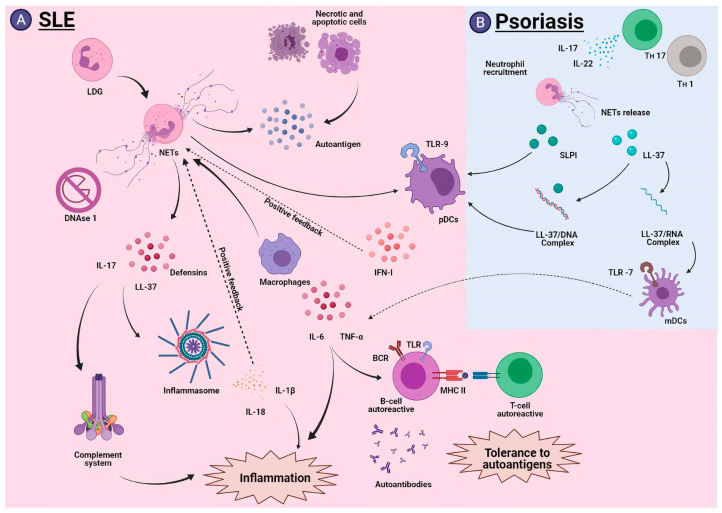
NETs in SLE and psoriasis. (**A**) During the development of SLE, LDGs are the main producers of NETs, which accumulate due to the DNAse 1 inhibition preventing their degradation. NETs have detrimental effects in SLE, since they release components such as LL-37 and IL-17 that activate the inflammasome and the complement system. NETs are internalized by macrophages, which release proinflammatory cytokines that lead to activation of autoreactive B cells and subsequent presentation to T cells and tolerance to autoantigens. Finally, NETs activate pDCs efficiently and stimulate IFN-I production, generating positive feedback to release more NETs. (**B**) Th17 cells are the main mediators of psoriasis by secreting IL-17; they promote neutrophil recruitment, and subsequently NET release. Their components stimulate the activation of pDCs directly (SLPI) or by DNA complexes (LL-37), to produce more IFN-I. In addition, LL-37 can form RNA complexes that promote the activation of mDCs and exacerbate the inflammatory process. SLE, systemic lupus erythematosus; LDG, low-density granulocytes; NET, neutrophil extracellular trap; LL-37, cathelicidin; IL-17, interleukin-17; pDC, plasmacytoid dendritic cells; IFN-I, type I interferon; SLPI, secretory leukocyte protease inhibitor; mDC, myeloid dendritic cells; mDC, myeloid dendritic cells. Created with BioRender.com.

SLE is a complex disease of autoimmune etiology that can affect multiple healthy organs throughout the body; it manifests itself mainly in skin, joints, kidneys and brain. The SLE is characterized by the production of autoantibodies against nuclear antigens and associated proteins, especially against components associated with NETs (Figure 5A). These autoantibodies generate the activation of an exacerbated immune response, which causes systemic inflammation, accompanied by immune-complex accumulation, derived from the failed autoantigen clearance of necrotic and apoptotic cells [126,127].

Some studies have shown that NETs are responsible for a large amount of autoantigens released during the SLE development, and that patients with this disease have an inefficient NET degradation due to the blocking of DNasa1 functions by inhibitors [128,129,130]. This promotes the activation of the complement system, generating an inflammatory response and tissue damage. NETs are known to be good activators of the plasmacytoid dendritic cells (pDC) through TLR-9 activation; this in turn causes the production of large amounts of type I interferon (IFN-I), and consequently, it stimulates other neutrophils to produce more NETs [131,132,133,134,135].

Studies have found that patients with SLE show an increase in LDG. These neutrophils produce NETs more efficiently than common neutrophils [136]. NETs generated by LDGs have a superior capacity to produce IFN-I due to the exacerbated ROS release through the activation of a STING-dependent pathway [137,138]. LDGs contain large amounts of autoantigens, and when they release their NETs, they also release components such as LL-37, IL-17 and α and β-defensins. These components lead to inflammasome activation through the interaction with the NLRP3 receptor [138,139]. This stimulates the cytokine production of IL-1β and IL-18, which successively induces NET formation, thus generating an exacerbated inflammatory response [139].

The NETs released by LDGs could regulate multiple cellular components involved in SLE immunity, both at the innate and adaptive level, such is the case of macrophages, which can internalize NETs and secrete the proinflammatory cytokines IL-6 and TNF-α in response; the production of these cytokines promotes differentiation into autoreactive B cells that produce autoantibodies, such as anti-LL37, exacerbating the pathology of SLE [140,141]. Additionally, NETs could interact with B cells through internalization by means of TLR or the BCRs themselves, thus achieving antigenic presentation in the context of MHC-II to CD4^+^ T cells with autoreactive capacity, which drives the production of cytokines that promote autoantibody production and more inflammatory components, which reflects the loss of tolerance to autoantibodies [142].

Therapeutic approaches have sought to improve the degradation of accumulated NETs, and thus support the treatment of SLE. In this regard, a peptide homologue derived from the spliceosomal protein U1-70K, known as P140, has been described. This peptide has the capacity to inhibit the release of NETs produced in SLE and thus reduce the amount of exposed autoantigens, reducing the effects that NETs exert on this pathology [143,144].

Recently, more NET-associated markers have been linked to SLE, such is the case of cell-free DNA (cfDNA), myeloperoxidase activity (MPO) and anti-MPO antibodies, which have been found to be higher in SLE patients, which may be useful to better understand the SLE pathogenesis and the development of new therapies [145]. Together, these studies demonstrate the key role NETs play in the pathogenesis of this disease.

### 5.2. Psoriasis

Psoriasis is an inflammatory, cutaneous and chronic disease of autoimmune origin, mediated by Th1 and Th17 cells. Its main characteristic is the appearance of lesions that affect the skin, especially arms, knees and scalp. Neutrophils are known to be among the first cellular elements to appear in erythematous lesions, and some studies describe an increase in the NET production by neutrophils, both at the level of injury and in peripheral blood. This increase has been correlated with the severity of the pathology [146,147]. Furthermore, IL-17 is known to be among the key cytokines in disease development, and increased levels of IL-17 have been reported in NET-associated psoriatic lesions [148]. This relationship reveals the role that NETs could play in the development of psoriasis (Figure 5B).

During NET generation, different components are released; among them is LL-37, which is associated with psoriasis because it has a cationic component that allows it to interact with the nucleic acids of the patient. This facilitates the formation of complexes that are deposited in skin lesions [149]. The LL-37/DNA complexes activate the TLR9 in pDC cells, which is manifested in the increased production of IFN-α [150], whereas the LL-37/RNA complexes activate the mDCs through a TLR7 pathway, which leads to the overproduction of proinflammatory cytokines, such as IL-6 and TNF-α [149]. These complexes have been correlated with early psoriasis development [151]. Additionally, another important component of NETs, called secretory leukocyte protease inhibitor (SLPI), has been detected, which can regulate the formation of new NETs, when colocalizing with the pDC. It forms a complex that is deposited in psoriatic lesions, and similar to the LL-37/DNA complex, it stimulates the TLR9, generating even greater amounts of IFN-I [152,153]. More recent studies have shown that NETs could contribute to the pathogenesis of psoriasis through a new mechanism independent of TLR7 or TLR9; this mechanism involves a crosstalk between TLR4 and IL-36R in psoriatic lesions, which could promote an inflammatory response, and highlight TLR4 as a possible new therapeutic target [154].

Finally, it has been reported that NETs could have an important effect in reducing susceptibility to infections by inducing the expression of β-defensin (HBD-2), a potent antimicrobial peptide produced by epidermal keratinocytes, which prevents infections in psoriatic lesion [146].

### 5.3. ANCA-Associated Small Vessel Vasculitis (AAV)

AAV is a systemic autoimmune disorder of unknown etiology characterized by the inflammation of small blood vessels in the arteries, arterioles and venules in various organs of the body, most notably the skin, kidneys and lungs [155,156,157,158]. The cutaneous clinical manifestations include palpable purpura, nodules, pustules, necrotic and ulcerative lesions, livedo reticularis and subcutaneous nodules [159]. Patients with AVV present anti-neutrophil cytoplasmic antibodies (ANCA) that target the main components of NETs, such as proteinase 3 and myeloperoxidase [160,161,162,163]. It has been suggested that these autoantibodies are mainly responsible for the disease, since they have been associated with the hyperactivation of neutrophils, maintenance of the inflammatory state through activation of the complement system and overproduction of NETs, which in turn has been associated with increased disease activity [163].

Other studies have proposed LDGs as the main NET producers in AAV due to their great capacity to produce them spontaneously, which renders this a positive feedback process that exacerbates the inflammatory process by allowing increased NET production [164]. ANCAs interact with various cellular components during the development of AAV, highlighting the interactions with neutrophils, eosinophils and monocytes that generate the exacerbation of the disease by inducing cellular cytotoxicity and tissue damage directly [165].

In additional studies on the pathogenesis of AAV, for example, it has been described that HMGB1, another component released during NETosis, has a potentiating function in the generation of ANCA-associated NETs, mediated by interaction with TLR2 and TLR4 [166]. In turn, it has been reported that NETs specifically activate the alternative complement pathway, which exacerbates the inflammatory process [162]; however, much further investigation is required to perfectly elucidate the pathogenesis of this disease.

## 6. NETs in Other Mucosal Surfaces

The role of NETs has been reported in diseases related to other barriers highly exposed to pathogens and external stimuli, such as the ocular surface and oral mucosa. The ocular surface comprises the cornea, conjunctiva and tear film, where antibodies, the complement system and innate immune cells participate in the defense against external stimuli [167]. The oral mucosa is among the most colonized surfaces by the microbiota, where cytokines, chemokines and salivary proteins, such as secretory IgA, IgG, lysozyme and mucins, as well as immune cells, such as neutrophils, macrophages and dendritic cells, maintain a balance between the oral commensals and the host [168,169].

### 6.1. Keratitis

Keratitis is the inflammation of the cornea, which can lead to loss of sight caused mainly by *P. aeruginosa* infection. Other common causative agents can be viruses (Herpes simplex, Varicella zoster and Adenovirus) or fungus. (*A. fumigatus, Aspergillus flavus, C. albicans, Fusarium solani, Acremonium, Penicillium*) [170,171,172]. In bacterial keratitis, neutrophils release NETs in response to the type three secretion system (T3SS) of *P. aeruginosa*, preventing their dissemination and eliminating the infection [170]. However, this effect depends on the type of strain of *P. aeruginosa*. NETs trap and limit the growth of invasive strains (e.g., PAO1 and 6294 strains), but cytotoxic strains (e.g., PA14 and 6077 strains) escape through the release of outer membrane vesicles (OMVs) to avoid NET binding [173]. Furthermore, NETs contribute to the corneal damage during this bacterial infection [174]. Likewise, it has been observed that there is release of NETs in in vitro models and in patients with fungal keratitis via the CR3 receptor, which induces ROS production, although further research is necessary to define the role of NETs in this infection [172,175,176,177].

### 6.2. Behçet’s Disease

Behçet’s disease (BD) is a systemic inflammatory disorder that mainly affects the uvea, retina, oral mucosa and genital cavity, causing recurrent ulcers, uveitis and vasculitis, as well as other cutaneous, gastrointestinal, respiratory and neurological manifestations [94]. A study by Mohanty et al. reported that the saliva from patients with BD showed impaired NET formation compared to healthy subjects [178]. However, other studies in BD patients indicate higher levels of NET release, cell free DNA and DNA–myeloperoxidase complexes, as well as spontaneous NET formation in comparison to healthy subjects. At the same time, this is associated with increased thrombin generation and vascular dysfunction, which indicates a detrimental role of NETs in BD pathology, through its contribution to vascular inflammation and to a prothrombotic state [179,180,181,182,183].

## 7. Conclusions

In this review, we analyzed the role NETs play in different inflammatory and infectious diseases that affect the epithelia. By exacerbation of a chronic inflammatory state, NETs cause a negative effect in different inflammatory and infectious diseases. On the other hand, several infectious agents have developed immune evasion mechanisms against the neutrophil response, such as NET formation, resulting in the dysfunction of the clearance of these microorganisms.

Different studies on IBD, COVID-19 and diseases associated with the respiratory tract report increased neutrophil recruitment and NET release, which promotes immunothrombosis, caused by the accumulation of DNA-peptide networks in the vasculature of the liver, lung and kidney. Moreover, in COPD, NETs promote the activation of lung-associated fibroblasts. Additionally, in infections caused by *N. gonorrhoeae* and *C. trachomatis*, NET release is inhibited through the secretion of factors such as thermonucleases, which degrade these DNA structures and proteases that block the activation pathways implicated in this mechanism.

Among the positive effects related to the presence of NETs is the antimicrobial effect in psoriasis, where they protect the host from major complications caused by opportunistic infections. A similar effect has been reported for the infections of the genitourinary tract, where NETs tend to eliminate the pathogens causing UTIs and candidiasis.

Although the negative effects of NET formation have been highlighted in various inflammatory and infectious diseases, further research is necessary to clarify the role of NETs both in the pathogenesis of inflammatory diseases, and in the eradication of infectious processes caused by different pathogens.

## Data Availability

Not applicable.
